# Draft genome sequencing of a mastitis-associated multidrug-resistant *Staphylococcus aureus* strain from buffalo milk

**DOI:** 10.1128/mra.00818-25

**Published:** 2025-09-25

**Authors:** Tanvir Shahriar, Mahmuda Nasrin Juthi, Naim Siddique, Kh. Yeashir Arafat, Md. Morshedur Rahman, Nashib Parajuli, M. Nazmul Hoque, Ziban Chandra Das

**Affiliations:** 1 Molecular Biology and Bioinformatics Laboratory, Department of Gynecology, Obstetrics and Reproductive Health, Gazipur Agricultural University (GAU) 198780, Gazipur, Bangladesh; The University of Arizona, Tucson, Arizona, USA

**Keywords:** multidrug resistance, *Staphylococcus aureus*, clinical mastitis, buffalo milk, genome sequencing

## Abstract

We report the draft genome of a multidrug-resistant *Staphylococcus aureus* strain, MBBL25_3BM, isolated from buffalo milk with clinical mastitis. The 2.72 Mbp genome, assembled into 57 contigs, underscores its pathogenic potential and poses a significant threat to mastitis management.

## ANNOUNCEMENT

*Staphylococcus aureus*, a major gram-positive pathogen, is typically associated with infections in both humans and animals, including mastitis in dairy cows and buffalo cows (1, 2). Because of multidrug resistance (MDR) capability and its zoonotic potential, *S. aureus* is of major concern (3, 4). This study reports draft genome sequencing of an *S. aureus* isolate recovered from clinical mastitis (CM)-affected buffalo cow milk, diagnosed through the California mastitis test (5), in the Bogura district (24.85° N, 89.37° E) of Bangladesh.

**Fig 1:**
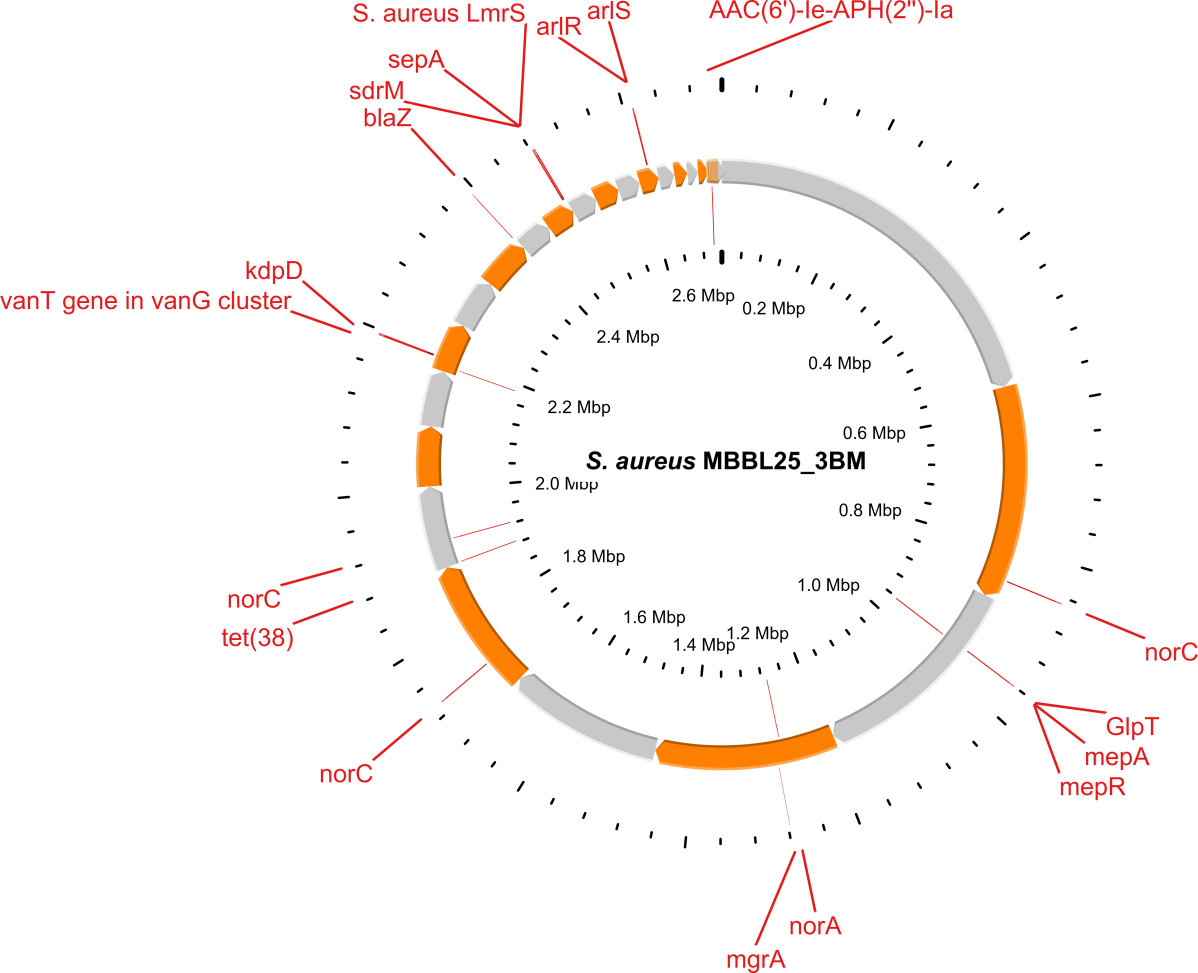
*S. aureus* MBBL25_3BM circular genome representing antibiotic resistance genes (ARGs). The orange and gray arrows in the middle represent the open reading frames (ORFs) of the genome. The circular genomic map was created using the Proksee server (https://proksee.ca/).

Milk samples were obtained following the standards set by the National Mastitis Council in 2014 (6). Approximately 100 µL of milk sample was inoculated immediately into 5 mL of nutrient broth (Biolife, Italy) and left for incubation overnight at 37°C (1, 2). The enriched broth (2 µL) was streaked onto Mannitol Salt Agar (MSA, Oxoid, UK) and incubated at 37°C for 24 h. Characteristic golden-yellow pigmented colonies with mannitol fermentation were suspected to be *S. aureus* colonies, which were sub-cultured onto MSA for purification. Gram staining (gram-positive clustered cocci), colony morphology, coagulase, and catalase tests were performed for phenotypic identification (7, 8). The isolate was identified as an MDR *S. aureus* isolate based on its phenotypic resistance to cefoxitin, ampicillin, oxacillin, vancomycin, colistin sulfate, and ciprofloxacin, determined through disc diffusion assay in accordance with (9) standards (9). Species-level identification of the isolate was confirmed using the VITEK 2 Compact system v. 9.01 (10). Genomic DNA from a purified single MDR *S. aureus* isolate (MBBL25_3BM) was extracted from nutrient broth culture using the QIAamp DNA Mini Kit (QIAGEN, Germany) following the manufacturer’s protocol. Whole-genome sequencing libraries were constructed from 1 ng of DNA using the Nextera DNA Flex Kit (Illumina, USA). The libraries were sequenced on an Illumina MiSeq platform, employing a 2×250 bp paired-end read configuration (11). Raw reads (*N*=2,817,396) underwent trimming with Trimmomatic v.0.39 (12) and quality checking through FastQC v.0.11.7 (13). The draft genome was assembled using SPAdes v.4.2.0 (14) and annotated using the NCBI Prokaryotic Genome Annotation Pipeline v.6.10 (15). Genome completeness, antimicrobial resistance genes (ARGs), virulence factor genes (VFGs), plasmid, pathogenicity score, CRISPR arrays, and metabolic functions in the draft genome were predicted using CheckM v.1.2.4 (16, 17, 17), VFDB v.6.0 (18), PlasmidFinder v.2.0.1 (19), PathogenFinder v.2 (20), CRISPRCasFinder v.2.0.3 (21), and RAST v.2.0 server (22), respectively. In all analyzes, default parameters were used unless otherwise specified. The features of the draft genome are presented in Table 1 and Fig. 1. We predicted 14 ARGs, 65 VFGs, 5 CRISPR arrays, and 359 metabolic features in the draft genome. However, no plasmid replicon was predicted in the draft genome. These findings underscore the significant involvement of *S. aureus* in bubaline mastitis and offer valuable insights into its antimicrobial resistance and virulence, aiding informed decisions on mastitis management in the buffalo farms.

**TABLE 1:** Genomic features of *S. aureus* strain MBBL25_3BM isolated from buffalo milk with CM

Genome feature	MBBL25_3BM
GenBank accession	JBPQCZ000000000
Assembled genome Size (bp)	2,716,917
GC content (%)	32.5
Coverage (x)	32
Genome completeness (%)	98.72
CheckM contamination (%)	0.45
BioSample accession	SAMN49720648
SRA accession	SRR34304564
*N_50_* value (bp)	267,403
Number of contigs	57
Genes (total)	2,711
Coding sequences (CDSs)	2,649
Genes (coding)	2,586
CDSs (with protein)	2,586
Genes (RNA)	62
rRNAs	2, 2, 1 (5S, 16S, 23S)
Complete rRNAs	1, 1 (5S, 23S)
tRNAs	53
ncRNAs	4
Pseudo genes	63
CDSs (without protein)	63
Pseudo genes (ambiguous residues)	0 of 63
Pseudo genes (frameshifted)	27 of 63
Pseudo genes (incomplete)	33 of 63
Pseudo genes (internal stop)	12 of 63
Pseudo genes (multiple problems)	Nine of 63
CRISPR arrays	5
Number of ARGs	14
Plasmid replicon	None
Number of virulence gene	65
Number of subsystems (metabolic features)	359
PathogenFinder score	0.986
Matched with pathogenic families	492

## Data Availability

The whole genome shotgun project of the *Staphylococcus aureus* strain MBBL25_3BM has been deposited in GenBank and the NCBI Sequence Read Archive (SRA) under BioProject accession number PRJNA1284368. The versions described in this paper is version JBPQCZ000000000.1.
